# B-cell depletion in SLE: clinical and trial experience with rituximab and ocrelizumab and implications for study design

**DOI:** 10.1186/ar3910

**Published:** 2013-02-11

**Authors:** Venkat Reddy, David Jayne, David Close, David Isenberg

**Affiliations:** 1 Centre for Rheumatology, The Rayne Building, 4th Floor, Room 424, 5 University Street, London WC1E 6JF, UK; 2 Department of Medicine, Cambridge University Hospitals NHS Foundation Trust, Cambridge CB2 0QQ, UK; 3 Inflammation and Autoimmunity, MedImmune Limited, Milstein Building, Granta Park, Cambridge CB21 6GH, UK

## Abstract

B cells are believed to be central to the disease process in systemic lupus erythematosus (SLE), making them a target for new therapeutic intervention. In recent years there have been many publications regarding the experience in SLE of B-cell depletion utilising rituximab, an anti-CD20 mAb that temporarily depletes B cells, reporting promising results in uncontrolled open studies and in routine clinical use. However, the two large randomised controlled trials in extra-renal lupus (EXPLORER study) and lupus nephritis (LUNAR study) failed to achieve their primary endpoints. Based on the clinical experience with rituximab this failure was somewhat unexpected and raised a number of questions and concerns, not only into the true level of benefit of B-cell depletion in a broad population but also how to test the true level of effectiveness of an investigational agent as we seek to improve the design of therapeutic trials in SLE. A better understanding of what went wrong in these trials is essential to elucidate the underlying reasons for the disparate observations noted in open studies and controlled trials. In this review, we focus on various factors that may affect the ability to accurately and confidently establish the level of treatment effect of the investigational agent, in this case rituximab, in the two studies and explore hurdles faced in the randomised controlled trials investigating the efficacy of ocrelizumab, the humanised anti-CD20 mAb, in SLE. Further, based on the lessons learned from the clinical trials, we make suggestions that could be implemented in future clinical trial design to overcome the hurdles faced.

## Background

B cells have been targeted in the treatment of systemic lupus erythematosus (SLE) and rheumatoid arthritis (RA) owing to the central role they play in the pathogenesis of these disorders. These cells play a critical role in host defence through their maturation into antibody-secreting plasma cells, secretion of proinflammatory cytokines, antigen presentation and co-stimulatory support for T cells. However, dysfunctional recognition of self-antigens as nonself-antigens results in autoantibody production, sustained by plasma cells derived from the B-cell lineage that survive for prolonged periods in the lymphoid tissues. B cells also participate in inflammatory reactions through antibody-independent mechanisms by acting as antigen-presenting cells and co-stimulation of T cells and other inflammatory cell types, although as yet there are no validated biomarkers that distinguish pathogenic from protective B-cell subsets. Reagents that specifically target pathogenic B-cell subsets are therefore not likely to be available in the near future. This reality provides the rationale for targeting B cells in patients with SLE, RA and other autoimmune diseases [1-5].

B-cell-targeted immunotherapy was initially developed for the treatment of B-cell-related malignancies, which are associated with poor prognosis despite aggressive cytotoxic therapies. Of the many surface-expressed antigens on B cells studied as possible targets, CD20 - a transmembrane phosphoprotein expressed in normal B cells as well as 90% of lymphomas - is not shed or modulated, making it an attractive target. In 1994, Reff and colleagues reported a major (95%) and sustained (up to 90 days) B-cell depletion using a murine mAb (2B8) that targeted CD20 on B cells in nonhuman primates [6]. In 1997, a landmark study reported on both the safety and efficacy of rituximab, a chimeric (mouse-human) mAb directed against CD20, for the treatment of relapsed, refractory low-grade or follicular lymphoma [7]. In November 1997, rituximab was licensed for this indication. Rituximab is now a part of the standard therapeutic regimen in the management of B-cell malignancies and remains among the most successful therapeutic mAbs. Interestingly, the response rate is variable amongst individuals with the same histological type of lymphoma as well as the overall response rate between different histological types [8]. This suggests that B-cell depletion is not uniform across patients or indeed diseases for reasons yet to be fully understood, but Fcγ receptor function appears important with enhanced Fcγ receptor IIb expression being associated with reduced rituximab efficacy in lymphoma [9]. Intriguingly, polymorphisms of this receptor are associated with SLE, although their precise role in the disease and potential for targeted therapeutic intervention is not understood.

In 1999, Professor Edwards' group at University College London treated a small number of patients with refractory RA using rituximab, having been encouraged by the safety and efficacy profile of induced transient depletion of B cells in haematological malignancies. This study and subsequent studies of rituximab in RA, including a large phase II randomised controlled trial, indicated that the treatment was potentially safe and effective [10-13]. The regimen in these studies utilised two doses (1,000 mg) of rituximab given 2 weeks apart, with premedication including a single 100 mg intravenous dose of methylprednisolone and 10 mg chlorphenamine. In the original study, patients also received a course of high-dose prednisolone (60 mg for up to 3 weeks and then tapering over the next 3 to 4 weeks or maintaining at 5 mg a day). Responding patients were retreated at or just before predicted relapse. Initially, intravenous cyclophosphamide was used to accompany the rituximab [10]. The phase II study showed that cyclophosphamide could be replaced by methotrexate or rituximab on its own, although the response rates were better when rituximab was used in combination with methotrexate. Further, the assigned dose of prednisolone was reduced to 60 mg/day oral prednisone on day 2 and days 4 to 7 and 30 mg/day oral prednisone on days 8 to 14 [11].

## Clinical experience of rituximab in SLE

The first open, uncontrolled study of rituximab for patients with SLE, by Professor Isenberg's group at University College London, showed improvements in both clinical and laboratory features of disease following treatment with rituximab in refractory SLE [14]; these observations have been supported by the publication of many other similar open, nonrandomised studies [15-19] (Table 1). The University College London regimen employed premedication with 100 mg intravenous methylprednisolone in addition to 750 mg low-dose intravenous cyclophosphamide (for renal manifestations) 1 day prior to the first of two doses of rituximab, given 2 weeks apart. More recently just one dose of cyclophosphamide has been used, and any subsequent need for immunosuppressive therapy is adjusted based on the merits of clinical response and disease manifestation activity that can be assessed using well-validated tools such as the British Isles Lupus Assessment Group (BILAG) 2004 index (for example, using the BLIPS computer software program; LIMATHON, Sheffield, UK).

**Table 1:** Reported efficacy of rituximab in nonrandomised trials of systemic lupus erythematosus

Study	**Rituximab regimen**	**Organ-specific disease**	**Number of patients/follow-up (months)**	**Method of assessment (mean disease activity score before/after B-cell depletion)**
Anolik and colleagues [64]; Looney and colleagues [26]	Variable	No (7 LN)	17/12	SLAM improved in patients achieving effective B-cell depletion (6.8/5.2)
Leandro and colleagues [15]^b^	2-dose	No (17/19 LN)	19/6	BILAG (13.9/5)
Vigna-Perez and colleagues [65]	2-dose	Yes, LN	22/3	Mexico-SLEDAI (10.8/6.8)
Cambridge and colleagues [21]^b^	2-dose	No (12/15 LN)	15/6	BILAG
Tamimoto and colleagues [66]	Variable	No (4/8LN)	8	SLEDAI (17.6/7.3)
Tokunaga and colleagues [28]	Variable	Yes, NPSLE	10/7 to 45	Neurological parameters (GCS)
Tanaka and colleagues [67]	2-dose	No (6LN)	14/7	BILAG (12.5/7.1)
Ng and colleagues [17]^b^	2-dose	No (21 LN)	32/39	BILAG (13/5)
Reynolds and colleagues [45]	Variable	No	11/10	BILAG (median reduction of 7.5)
Li and colleagues [68],	2-dose	Yes, LN	19/12	SLEDAI (9.2/2.5)
Lu and colleagues [69]^b^	2-dose	No (33/45 LN)	45/39.6	BILAG (12/5)
Pepper and colleagues [56]	2-dose + MMF maintenance	Yes, LN	20/12	Renal parameters improved in 14/18 at 12 months
Catapano and colleagues [19]	4-dose (15) or2-dose + CYC (16)	No (11 LN)	31/30	BILAG (14.5/3.5 at 24 months)
Sfikakis and colleagues [70]	4-dose	Yes, LN	10/12	Renal parameters
Gottenberg and colleagues [71]	4-dose	No (4 LN)	13/8.3	SLEDAI (8/2)
Smith and colleagues [18]	4-dose, retreated with 2-dose	No	11/24	BILAG (14/2)
Gunnarsson and colleagues [72]	4-dose	Yes, LN	7/6	SLEDAI (15/3)
Galarza and colleagues [73]	4-dose	No	43/12	SLEDAI (12.5/4.5)
Jonsdottir and colleagues [74]	4-dose	No (10 LN)	16/27	SLEDAI (12.1/4.7)
Lindholm and colleagues [75]	4-dose	No (17 LN)	29/22	Renal parameters
Sutter and colleagues [76]	4-dose	No	12	SLEDAI (9/5)
Boletis and colleagues [77]	4-dose	Yes, LN	10/38	Renal parameters
Melander and colleagues [78]	4-dose regimen (10 retreated)	Yes, LN	20/22	12/20 improved

BILAG, British Isles Lupus Assessment Group; CYC, cyclophosphamide; GCS, Glasgow Coma Scale; MMF, mycophenolate mofetil; SLAM, systemic lupus activity measure; LN, lupus nephritis; NPSLE, neuropsychiatric systemic lupus erythematosus; SLEDAI, Systemic Lupus Erythematosus Disease Activity Index. ^a^Randomised controlled trial. ^b^Same cohort in these studies.

Appreciating this robust clinical management focused on the individual patient - potentially involving multi-disciplinary expert opinion, including rheumatologists, dermatologists and renal physicians - is important when comparing the results with those from large multicentre randomised controlled trials with variable quality observations in a broad population.

Worthy of note is that the indication for rituximab at Professor Isenberg's centre is a combination of active disease (renal or nonrenal) (assessed by the BILAG 2004 index) poorly controlled despite at least two standard immunosuppressive agents (not including corticosteroids) used for sufficient time at optimal doses. To date, 100 patients have been treated at University College London with at least one cycle of rituximab and more than 30 patients have received repeated treatment. Although involving only small numbers, the observations from repeating the regimen showed that improvements in disease, including remission rates, were sustained in patients who responded to the initial treatment [20]. This same group has previously demonstrated following B-cell depletion therapy (BCDT) that anti-double-stranded DNA (anti-dsDNA) and anti-nucleosome antibodies reduce to 30 to 40% of baseline, whereas other autoantibodies such as anti-Ro and antibodies to pneumococcal polysaccharide (protective) remain unaltered. This observation would suggest that rapidly proliferating clones of B cells may give rise to short-lived plasma cells that produce these anti-dsDNA, anti-cardiolipin and anti-nucleosome antibodies and appear preferentially affected by BCDT [21], whereas other autoantibodies such as anti-Ro and anti-RNP or protective antibodies, which develop following immunisation and are thought to be produced by long-lived plasma cells, remain unaltered.

In line with this experience, anti-dsDNA antibody levels tend to fall but not to normalise and these antibodies are probably produced by a combination of short-lived and long-lived plasma cells. Similar to these findings, a *post-hoc *analysis of the EXPLORER trial focusing on the biological effects of rituximab revealed a significant reduction in the levels of anti-dsDNA and anti-cardiolipin antibodies and a significant increase in complement levels and serum BAFF in the rituximab-treated group versus placebo. Analysis of the repopulation dynamics of subsets of B cells identified naïve cells as the primary phenotype detected first in circulation; however, the phenotype analysis was limited in that CD27^- ^memory cells were not examined in this study [22]. The changes in biological effects did not translate into clinical benefits at 1 year. Whether a long-term follow up with more detailed phenotype analysis at various time points would help predict response to rituximab therapy is not known. However, designing clinical trials to define the precise relationship between the biological effects that occur following BCDT and the clinical response in the long term (typically, 2 to 5 years) would be met with the potential challenge of maintaining remission in the placebo group with conventional immunosuppressants alone. The effects extend to global disease control including an improvement in lipid profile [23], but such benefits are not necessarily captured in randomised controlled trials with a short duration of follow-up.

Recently, following the approach by a group at Imperial College (see later) in a pilot study, eight patients with active disease were treated at diagnosis with rituximab in an attempt to avoid the use of corticosteroids. Using this approach it was possible to reduce the cumulative dose of steroids substantially in five of the eight patients [24], a major long-term advantage.

A recent review of the rituximab experience in approximately 200 patients with refractory SLE, from open studies and real clinical experience, indicated that many would respond at least partially to B-cell depletion [25]. Differences in determining endpoints for these studies make it difficult to establish formal median and range of improvements. In a phase I/II dose-escalation trial of the safety and efficacy of rituximab in addition to ongoing therapy in 18 patients with SLE, three dosing regimens of rituximab were studied as follows: six patients received a low dose, a single infusion of 100 mg/m^2^; six patients received an intermediate dose, a single infusion of 375 mg/m^2^; and five patients received a high dose, four infusions of 375 mg/m^2 ^administered 1 week apart. There was a significant improvement in the disease activity, as measured by systemic lupus activity measure scores, in all patients by 2 months, which persisted at 12 months regardless of a change in anti-dsDNA antibody and complement levels. Six of 17 patients developed human anti-chimeric antibodies, resulting in reduced serum rituximab levels and inefficient B-cell depletion and less impressive efficacy. Importantly, there were no significant adverse events [26]. The UK-BIOGEAS registry study of 164 patients with refractory or relapsing lupus nephritis reported a 67% partial or complete response rate to rituximab using standardised response criteria [27].

Clinicians therefore continue to use rituximab for refractory lupus nephritis as well as nonrenal manifestations including haematological, skin and central nervous system manifestations where clinically useful responses have been reported [28,29]. There is thus extensive nonrandomised and retrospective experience of rituximab in the treatment of refractory SLE. A role for rituximab for this indication is supported by the consistency of the reports of improvement but differences in regimens, concomitant medications and endpoints remain, making it difficult to assess the extent of effectiveness of B-cell depletion accurately. Additionally, there is uncertainty as to how to reduce relapse risk after rituximab, and an unqualified recommendation for rituximab in refractory SLE will require higher quality evidence.

## Safety and efficacy in clinical trials

To evaluate the safety and efficacy of rituximab in SLE in a clinical trial setting, two double-blind, randomised, placebo-controlled trials (DBRCTs) investigating renal (LUNAR study) and nonrenal (EXPLORER study) manifestations were undertaken (Table 2). Both trials addressed the hypothesis that the addition of rituximab to the standard of care, corticosteroids and immunosuppressants was superior to addition of placebo for the control of SLE activity.

**Table 2:** Summary of the randomised-controlled trials of rituximab therapy in systemic lupus erythematosus

Study	Rituximab regimen	Concomitant therapy	Endpoints	Results
LUNAR	Randomised 1:1 to receive either rituximab or placebo on days 1, 15, 168, and 182	MMF and corticosteroids	Primary: (i) % patients with complete or partial renal responses at week 52. Secondary: (ii) patients with BL UPCR >3 to UPCR <1; (iii) % change from BL in anti-dsDNA; and (iv) mean change from BL in C3 (mg/dl)	(i) and (ii) no significant difference; (iii) placebo (50%) and rituximab (69%) (*P *<0.01); and (iv) placebo (25.9%) and rituximab (37.5%) (*P *<0.03). % patients requiring a new immunosuppressive agent placebo (11.1%) and rituximab (1.4%)
EXPLORER	Randomised 1:2 to receive placebo or rituximab, methyl prednisolone 100 mg and acetaminophen and diphenhydramine or placebo on days 1, 15, 168, and 182	Usual dose prednisolone and either azathioprine 100 to 250 mg/day, MMF 1 to 4 g/day or MTX 7.5 to 27.5 mg/week, and additional prednisolone (0.5 mg/kg, 0.75 mg/kg, or 1.0 mg/kg), tapered beginning on day 16 to a dosage of 10 mg/day over 10 weeks and 5 mg/day by week 52	Primary: effect of placebo or rituximab in achieving and maintaining a major, partial or no response at week 52 in each of the eight BILAG index organ system scores. Secondary: described earlier	Primary EP: major clinical response 15.9% vs. 12.4% and PCR 12.5% vs. 17.2% for placebo and rituximab, respectively. In the African American/Hispanic group: major clinical response 9.4% vs. 13.8% and PCR 6.3% vs. 20.0% for placebo and rituximab, respectively
Li and colleagues [68]	Randomised to receive either rituximab or a combination of rituximab and cyclophosphamide 750 mg on day 1 and day 15, followed by intravenous methylprednisolone 250 mg and oral prednisolone 30 mg from day 2 to day 5, then 0.5 mg/kg for 4 weeks and then reducing the dose by 5 mg every 2 weeks to 5 mg/day	Other medications were stopped except for hydroxychloroquine, oral prednisolone and statins. All patients also received angiotensin-converting enzymes inhibitors	Primary: in each of the groups, % patients with complete response at week 48. Secondary: % patients with partial response; and duration of complete CD19^+ ^B-lymphocyte depletion, histological assessment, adverse effects or death at week 48	Primary EP: no significant difference between the two groups. Overall, at week 48, 21% had a complete response, 58% achieved partial response, 11% remained the same and 11% worsened. Secondary EP: 42% patients achieved a complete response; 95% achieved effective depletion; no significant difference in the proportion of patients achieving a complete depletion at weeks 4, 8, 24 and 48 between the two groups except at week 2; a significant improvement in mean serum albumin levels (28.1 to 39.4), changes in the concentration of serum C3 (0.55 to 0.85), dsDNA antibody (693 to 8) and immunoglobulins. At week 48, the urinary protein excretion improved and there was an improvement in the ESR (62.1 to 30) and SLEDAI (9.2 to 2.5)

BL, baseline; EP, endpoint; ESR, erythrocyte sedimentation rate; MMF, mycophenolate mofetil; MTX, methotrexate; PCR, partial clinical response; SLEDAI, Systemic Lupus Erythematosus Disease Activity Index; UPCR, urine protein creatinine ratio.

**Figure 1:**
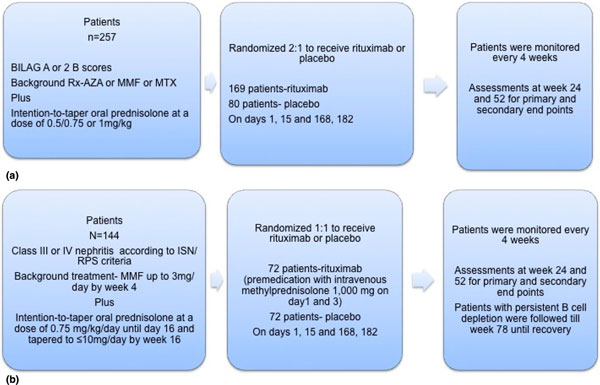
**Treatment protocol of the BELONG study**. AZT, azathioprene; CYC, cyclophosphamide; EL, EUROLUPUS; LN, lupus nephritis; MMF, mycophenolate mofetil; OCR, ocrelizumab; ORR, overall renal response; PBO, placebo.

In the EXPLORER study, the safety and efficacy of rituximab in moderate-to-severe active nonrenal SLE was evaluated [30] (Figure 1). This study included 257 patients with ≥1 BILAG A score (>50% of patients at entry) or ≥2 BILAG B scores despite ongoing stable-dose immunosuppressant therapy with either azathioprine (100 to 250 mg/day), mycophenolate (1 to 4 g/day) or methotrexate (7.5 to 25 mg/week), which was continued during the trial. Background immunosuppressive therapy was evenly distributed. A key feature of treatment in this study was the additional course of high-dose corticosteroids patients received early in the study. Corticosteroids were given at initial doses of 0.5 mg/kg, 0.75 mg/kg or 1 mg/kg depending on severity (by BILAG score) and type of disease manifestations, followed by a taper regimen. Of the overall population, >50% were classed as steroid dependent, and ≥60% of patients received an average 45.9 ± 16.4 mg prednisolone and then attempted to reduce to a target dose of <10 mg/day over the 10-week taper period and ≤5 mg/day at week 52.

Patients were randomised at a ratio of 2:1 to receive rituximab (1,000 mg) or placebo. Eighty-eight patients received placebo and 169 patients received rituximab (two doses given 14 days apart) on days 1, 15, 168, and 182. The majority (≥50%) of patients in both groups had musculoskeletal and mucocutaneous disease.

The primary endpoint of the EXPLORER study was stringent, with complete and partial response definitions as follows.

To classify as a complete/major response, at week 24 an improvement in all organ systems with a BILAG C score or better was required. Further, this response was to be sustained at week 52, without experiencing a severe or moderate/severe flare during the period to week 24 and week 52, respectively. A severe flare was defined as a BILAG A score or as two new domains with BILAG B scores [31].

Patients were considered to have attained a partial response if: there was an improvement in all organ systems with a BILAG C score or better, which was sustained for 16 consecutive weeks; a BILAG B score in no more than one organ system at week 24 without a new BILAG A or BILAG B score to week 52 was achieved; and, at week 24, no more than two BILAG B scores were achieved without new BILAG A or BILAG B scores provided the baseline BILAG score was one A score plus ≥2 B scores, ≥2 A scores, or ≥4 B scores.

The secondary endpoints included the time-adjusted area under the curve minus the baseline BILAG score over 52 weeks, the proportion of patients who achieved a major and partial clinical response, the proportion of patients who achieved a BILAG C score in all organ systems at week 24, the time to the first moderate to severe disease flare, improvement in quality of life as measured by the Lupus Quality of Life, and the proportion of patients who achieved a major clinical response with a prednisolone dose <10 mg/day from week 24 to week 52. In addition, serological activity parameters including levels of autoantibodies, complement, immunoglobulins, T-cell and B-cell counts and human anti-chimeric antibody were monitored.

In the intent-to-treat analysis of 257 patients, approximately 70% of patients completed the study in both arms and the safety and tolerability was similar in both groups. There was no difference between the addition of placebo and rituximab to the standard of care in the primary and secondary efficacy endpoints, including the BILAG-defined response, in terms of both area under the curve and other analyses.

A preplanned subgroup analysis, however, detected a beneficial effect of rituximab in the primary endpoint in the African American and Hispanic patients, a major clinical response in 13.8% and a partial response in 20% when compared with 9.4% and 6%, respectively. Notably, these patients had more active disease and more refractory disease as previously reported [32]. There were significant biological effects in the rituximab-treated group, with greater falls in anti-dsDNA levels and rises in complement levels compared with placebo. Interestingly, up to 9.5% of patients did not achieve complete B-cell depletion, but analysis without these patients did not change the primary outcome. This phenomenon has been observed in autoimmune prone mice [33,34]. A recent study investigating the role of highly sensitive flow cytometry detected a correlation between clinical response and B-cell numbers [35].

**Figure 2:**
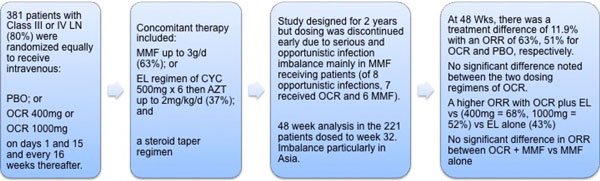
**Treatment protocols of the EXPLORER and LUNAR studies**. **(a) **EXPLORER study. **(b) **LUNAR study. BILAG, British Isles Lupus Assessment Group; ISN/RPS, International Society of Nephrology/Renal Pathology Society; MMF, mycophenolate mofetil; MTX, methotrexate; Rx-AZA, treatment with azathioprine.

The LUNAR study investigated the safety and efficacy of 2 × 1,000 mg rituximab, at both 0 and 6 months, as compared with placebo in addition to background therapy with high-dose glucocorticoids and mycophenolate mofetil 3 g/day in 144 patients with proliferative lupus nephritis, classes III and IV (Figure 2).

The primary endpoint of the study was the proportion of patients with a complete or partial remission of nephritis at 12 months. Complete response was defined as, at week 52: serum creatinine improving from abnormal to normal level or from normal to ≤115% of baseline normal; a fall in the urine protein-creatinine ratio to <0.5; and urine sediment containing <5 red blood cells in a high-power field without casts. Patients who did not meet complete response were considered to have achieved a partial response if: serum creatinine reduced to ≤115% of abnormal baseline; the number of red blood cells/high-power field reduced to ≤50% baseline without red blood cell casts; and a reduction in urine protein-creatinine ratio from ≥3.0 to ≤3.0 or to <1 from ≤3.0.

The secondary endpoints were: complete renal response sustained from week 24 to week 52; time to first complete renal response; and, at week 52, the urine protein-creatinine ratio improving from >3 to <1, the time-adjusted area under the curve minus the BILAG global score, and a change in the physical function of SF-36 health survey. As in the EXPLORER study, serological indices, human anti-chimeric antibodies and B-cell depletion were monitored.

The response rates for rituximab and placebo were 26% and 30% for complete renal response and 30% and 15% for partial renal response, respectively. At week 52, more patients in the placebo arm (8 patients vs. 0 patients) received rescue cyclophosphamide therapy. Improvement in proteinuria was 32% and 9% for rituximab and placebo, respectively. Analogous to the findings in the EXPLORER study and the ALMS trial, a greater proportion of black patients responded favourably, although this was not statistically significant. There was a greater reduction in anti-dsDNA levels in the rituximab-treated group. Whether the response noted in patients of African ancestry is attributable to the disease severity alone or whether there are potential differences in B-cell responsiveness to rituximab therapy in these patients is as yet unclear. In this respect, it is worth noting that ethnicity might influence the clinical response to treatment even with conventional immunosuppressants as noted in the ALMS study. Our own data (D Isenberg, unpublished observations) has not indicated a clearly different outcome at 12 months post BCDT comparing Caucasians, Afro-Caribbean or Asian patients. Drawing any firm conclusions based on the disease severity alone would therefore be difficult.

However, overall this was a negative study in that there was no significant difference between the rituximab group and the placebo group. The absolute difference in response was 11%, with 54% and 43% responding in the rituximab and placebo groups, respectively [36]. This value was less than the planned 23% difference, which in retrospect looks over-optimistic especially considering the analysis at only 12 months in this population. Again, differences in serological markers between groups were found and a subsequent analysis found greater falls of proteinuria in the rituximab group. More African patients in the rituximab group responded and cyclophosphamide rescue was required more frequently in the placebo group. Therefore, despite some clear signals of efficacy and safety, this study did not meet its primary or secondary endpoints.

Why did these two DBRCTs fail to meet their endpoints? As discussed earlier, there are several confounding factors that may have masked the ability to accurately quantify any significant clinically meaningful beneficial effects of rituximab (Table 3), perhaps the most important being the aggressive background immunosuppressive therapy in the placebo and rituximab-treated groups. High-dose corticosteroids, in particular, may have prevented the full extent of efficacy of rituximab becoming evident, a factor that warrants due consideration in the design of future clinical trials for any investigational agent. The dilemma for trial designers is how rapidly to reduce glucocorticoid in patients with organ-threatening SLE. Trials with duration beyond 12 months would have greater chance of demonstrating the specific treatment effect that could be attributed to rituximab if corticosteroids are reduced to low levels during the first 6 months. Corticosteroid dosing could also be included in the threshold for response in trial endpoints. For example, standard treatment should allow low-dose prednisolone and the proportion of patients requiring >7.5 mg/day prednisolone could be classed as a failure. In the open studies, response was defined with such stringent criteria. Furthermore, applying such criteria would not detect organ-specific improvement; for example, a significant sustained improvement in a severe haematological abnormality but concurrent minor or moderate flare in skin or mucocutaneous disease would be classified as a failure.

**Table 3:** Potential explanations for the apparent discrepancy in clinical response reported in clinical experience and DBRCTs

	Clinical experience	Randomised controlled trials
Disease activity	Refractory to conventional immunosuppressants	Rituximab was used as an add-on therapy to background immunosuppressants
	Favourable response reported in life-threatening cases, often including a range of organ-system involvement such as CNS manifestations, cytopenias and others	Life-threatening cases and those with CNS manifestations were not evaluated in controlled trials. This setting warrants a dedicated study
Clinical response	No defined pretreatment, therefore complete and partial responders might not be clearly distinguished	Predefined endpoints were stringent, perhaps driven by the impressive responses seen in clinical experience in an uncontrolled setting
	Improvement in one system alone might qualify for response, regardless of a flare or lack of response in another organ system	Predefined and usually stringent. For example, despite clinical response and steroid-sparing effect, a reduction in proteinuria that does not meet the predefined threshold would not qualify as complete/partial response
Background immunosuppressants	Flexibility in changes to background immunosuppressants including the dose of corticosteroids	Changes to or deviation with predefined background therapy would qualify as nonresponder
	Concomitant use of large dose of steroids is uncommon	Concomitant use of large dose of corticosteroids might have limited any beneficial effects of rituximab, the extent of which may be more restricted in such a setting than previously assumed
Rituximab dosing-regimen	Variable between reports	Predefined dosing regimen
Steroid tapering	Steroid-sparing effect is not a requirement to define response and therefore favourable response might be overestimated	Steroid dosing effect was included in the definition of clinical response
Adverse events	No standardised reporting of adverse events. Therefore, the true incidence of serious adverse events in clinical practice is not comparable with that reported in other uncontrolled studies or controlled clinical trials	Rituximab therapy appears to be safe as no there were no significant differences in serious adverse events when compared with standard-of-care treatment
Follow-up period	Not defined, therefore it is not known how many responders had sustained response in the long term	Predefined, therefore, unless long-term studies are undertaken, it would be difficult to detect the importance of effects seen at relatively short-term follow-up

CNS, central nervous system.

The planned efficacy margin in the LUNAR study was influenced by the 55% complete and partial response rate in the ALMS trial at 6 months using either mycophenolate or cyclophosphamide and corticosteroids. This suggested that 45% did not respond to standard of care; however, reasons for failure in the ALMS trial included death, severe adverse events, drug intolerance and patient/physician preference. One can estimate that true treatment failure was closer to 25% than 45%. A further factor in nephritis trials is the delayed response of the outcome measure, proteinuria, to reduction in histological activity in the kidney. The true time to remission of proteinuria is up to 2 years. Had the LUNAR trial aimed for a 12% efficacy difference and involved a 2-year duration, the study may have met its endpoint despite a small sample size.

One should also note that to date there is insufficient evidence to support the routine use of rituximab therapy for patients with specific neuropsychiatric manifestations. However, in a study of 10 patients with a range of neuropsychiatric manifestations (including cognitive dysfunction, psychosis and seizures) refractory to conventional immunosuppressants, including intravenous cyclophosphamide, there was a significant improvement, measured by the Systemic Lupus Erythematosus Disease Activity Index score at 28 days after treatment with rituximab, in all patients - and in five patients the response lasted for more than 1 year [28].

The other anti-CD20 mAb investigated in clinical trials for SLE is ocrelizumab (a humanised anti-CD20 mAb). In rheumatoid arthritis, ocrelizumab (two regimens used: 200 mg and 500 mg ×2 every 6 months) was effective in reducing signs and symptoms and joint damage when added to a stable dose of methotrexate [37,38]. However, a detailed analysis of results from four DBRCTs investigating the safety and efficacy of ocrelizumab for RA indicated that an increase in serious infections associated with ocrelizumab compared with placebo were dose dependent and occurred more frequently in Asia (particularly Japan) [39].

Two simultaneous clinical trials were initiated to study the safety and efficacy in lupus. Ocrelizumab was dosed differently from the RA and the rituximab SLE studies, at either 400 or 1,000 mg intravenously ×2 at entry with repeat, single dosing every 4 months. This regimen was designed to induce and maintain B-cell depletion throughout the trial periods. The BEGIN study for nonrenal SLE was cancelled early. The BELONG study for proliferative lupus nephritis compared 1,000 mg or 400 mg ocrelizumab at 1 day and 15 days, then repeated with a single dose every 4 months on a background of high-dose glucocorticoids and either mycophenolate mofetil or cyclophosphamide dosed according to the EUROLUPUS protocol (Figure 1). Although the study was designed to continue for to at least 2 years, the primary endpoint was the proportion of patients achieving partial or complete nephritis remission at 48 weeks. A total of 381 patients were recruited before the trial was stopped early due to an imbalance in the rate of serious infections in the ocrelizumab patients receiving mycophenolate. The 221 patients who had passed the 32-week treatment point were assessed. The absolute difference in renal response was 12%, with 63% and 51% for the combined ocrelizumab and placebo groups prospectively. However, it is worth noting that in the subgroup analysis there was a greater treatment effect of ocrelizumab when combined with the EUROLUPUS cyclophosphamide regime (renal response of 65.7% for ocrelizumab vs. 42.9% for EUROLUPUS alone) than with mycophenolate mofetil (renal response of 67.9% for ocrelizumab vs. 61.7% for mycophenolate alone), which was largely explained by a higher response rate in general with mycophenolate mofetil whilst perhaps again reflecting the outcome seen with rituximab in the LUNAR study [40] (Table 4).

**Table 4:** Safety and efficacy of ocrelizumab in lupus nephritis: design and results of the BELONG study

Patients and methods	Concomitant therapy	Endpoints	Results
A total of 381 patients with class III or class IV (80%) LN were randomised equally to receive either: placebo, OCR 400 mg or OCR 1,000 mg on days 1, 15 and every 16 weeks thereafter, >74% received three infusions and >50% received four infusions	In addition, either: MMF up to 3 g/day (63%); or EL (cyclophosphamide 500 mg ×6/2 weeks) followed by azathioprine 2 mg/kg up to 200 mg/day; and a steroid taper regimen - intravenous steroids: allowed up to 3 g by day 15, given in divided pulses), oral steroids: 0.5 to 0.75 mg/kg (≤60 mg/day) with taper to ≤10 mg over 10 weeks	Complete renal response: normal serum creatinine and ≤25% higher than baseline; urinary protein to creatinine ratio <0.5; inactive urinary sediment	In all modified intention-to-treat populations, there was a treatment difference of 12.2% with 54.7% vs. 66.9% for placebo (*n *= 75) and OCR (*n *= 148) groups, respectively
		Partial renal response: serum creatinine ≤25% above baseline value; and 50% improvement in the urine protein to creatinine ratio, and if baseline ratio >3.0 then a urine protein to creatinine ratio <3.0	ORR higher in OCR (400 mg) + EL (65.6%) and OCR (1,000 mg) + EL (74.2%) groups vs. placebo + EL (42.9%), ORR was similar in OCR+ MMF (67.9%) vs. placebo + MMF (61.7%)
		Nonresponse: not achieving either a complete or partial renal response. Patients who died or discontinued the study prior to week 48 (and had no renal data within 12 weeks of week 48) were considered nonresponders	≥50% reduction in urine protein-to-creatinine ratio occurred in 69.6% vs. 58.7 % for OCR and placebo groups, respectively
			Urine protein-to-creatinine ratio <0.5 was achieved in 39.9% vs. 37.3% for OCR and placebo, respectively
			Serious adverse effects imbalance appeared to be driven by the combination with MMF: OCR 400 mg (41.8%) compared with 1,000 mg OCR + MMF (24.1%) and placebo + MMF (21.3%). Serious adverse event rates in EL groups were not reported as higher in the OCR arms
			Serious infection imbalance appeared to be driven by the OCR combination with MMF. MMF groups: OCR 400 mg (32.9%) compared with 1,000 mg OCR (19%) and placebo + MMF (16.3%). EL groups: OCR 400 mg (12.8%) compared with 1,000 mg OCR (10.4%) and placebo + MMF (11.1%)

EL, EUROLUPUS regimen (cyclophosphamide followed by azathioprine); LN, lupus nephritis; MMF, mycophenolate mofetil; OCR, ocrelizumab; ORR, overall renal response.

Efficacy of the BCDT has also been demonstrated in another autoimmune condition, relapsing-remitting multiple sclerosis. A recent phase II randomised clinical trial investigating the safety and efficacy of ocrelizumab (given together with pre-infusion steroids only) in multiple sclerosis showed a significant reduction in neurological lesions compared with placebo as assessed by gadolinium-enhanced magnetic resonance images. Serious adverse events occurred in three of 55 patients receiving 2,000 mg ocrelizumab (one of 55 patients receiving 600 mg ocrelizumab, and two of 54 patients each in the placebo group and the IFNβ-1a group) [41]. These results also support the notion that treatment regimens of BCDT continue to have the potential to be safe in the wider context of treatment for chronic refractory autoimmune diseases.

Although not the principal focus of this review, it is notable in two trials involving >800 patients in each trial that belimumab (Benlysta), an anti-BLyS antibody, met its primary endpoint with a 10% and 14% absolute response difference over placebo [42,43]. The primary endpoint was a composite score, the SLE Responder Index, comprising a fall in Systemic Lupus Erythematosus Disease Activity Index of 4 points, no new BILAG A or B scores, and no change in the physician's global assessment. The comparisons were made at the start of the study, and at 52 or 76 weeks. These studies demonstrate: the need for larger trials looking for small but meaningful treatment effects; the potential efficacy of B-cell-targeted therapy; a similar magnitude of response to that seen in the LUNAR and BELONG studies, which collectively raises the question of defining a clinically meaningful treatment effect in SLE trials; and a new approach to defining a primary endpoint, the SLE Responder Index.

## Lessons learned so far and future clinical trial design - how to get it right?

The failure of clinical trials in SLE has introduced palpable uncertainty whilst providing some invaluable lessons regarding expectations for potential new therapies, carefully planned trial designs and appropriate endpoints for the particular agent/regimen in question. It is relevant to note that most preliminary data used rituximab for refractory SLE when standard agents had failed. This is in contrast to the randomised trials, which added rituximab on top of standard therapy for nonrefractory patients. Several factors specific to SLE increase the complexity in designing successful trials. RA is a less heterogeneous disease and is much better understood when compared with SLE and when arthritis is the main manifestation, despite the potential for other organs to be involved. Moreover, there exists a good deal more standardisation for clinical trials including validated endpoints - for example, Disease Activity Index, 28-joint Disease Activity Score. Conducting large-scale studies in a relatively short period of time is therefore possible - particularly as RA is more common and patient access is better, making statistically powered studies of relatively short duration feasible. For lupus, including nephritis, we are still some distance from achieving the same level of understanding and standardisation in the clinical trial setting.

In an attempt to improve the lupus patient's great unmet need, the European League Against Rheumatism has made a few suggestions to help researchers design successful trials [44]. The main points for the future design of clinical trials are to use strictly evaluated (a surrogate of therapeutic success against mortality or end-organ failure) outcome measures, including the disease activity indices, and to follow a standardised approach towards recording adverse events that could be used to measure benefit-to-risk ratios from interventions, comparable between trials. Increasingly important in future trials, when comparing the interventional drugs, is the real difference there may be in their potential to cause harm in the long term.

The aims of randomised controlled trials are to be defined to test robust hypotheses generated based on the available evidence from the open studies and clinical experience. Further, careful attention needs be paid when considering important factors, patient selection and sample size, the therapeutic agent or regimen and its potential effectiveness (and meaningful treatment delta vs. control), the disease outcome measures and disease activity indices, adequate follow-up and the adverse events (Tables 5 and 6). These variable factors contribute to a great element of uncertainty in predicting the probability of the success of clinical trial design in SLE.

**Table 5:** Adverse events reported in published studies^a ^during or after rituximab-induced B-cell depletion therapy

Infections	Pneumonia^b^
	Shingles^b^
	Thigh abscess, subcutaneous abscess
	Urinary tract infection
	Septicaemia
	*Psuedomonas *infection
	Staphyloccal abscess
	Streptococcal viridans infection
	Necrotising fasciitis
	Fatal histoplasmosis
Haematological	Neutropenia^b^
Pulmonary	Pneumonia
	Pulmonary haemorrhage
	Pulmonary embolism
	Respiratory failure^c^
	Breathlessness
Cardiac	Cardiac failure^c^
	Fatal pancarditis^c^
	Pericarditis
	Tachycardia
Neurological	Insomnia
	Transient ischaemic attack
Skin	Localised or widespread rash^b^
	Pruritis
	Urticaria
Miscellaneous	Infusion reactions^b^
	Serum sickness reaction
	Hypogammaglobulinaemia
	Anaphylaxis
	Deep vein thrombosis
	Dyspepsia
	Malaise
	Pyrexia
	Polyarthralgia

^a^See Table 1. ^b^Frequently reported adverse event. ^c^Life-threatening complications.

**Table 6:** Challenging areas in trial design and possible options

Patient selection and sample size
• Exclude seronegative patients
• Define the disease activity using a validated disease activity index
• Define refractory disease as either failure to respond to one or more immunosuppressants and an assigned dose of corticosteroids
• Ensure adequate sample size based on statistical power calculation to allow detection of even small therapeutic effects
• Allow for proportional representation of patients taking into account factors such as race, age, the duration of disease and type of organ involvement. For example, different histological types of nephritis may have variable sensitivity to B-cell depletion therapy
B-cell depletion
• Standardise the definition of adequate degree of B-cell depletion; for example, <5 cells/μl
The treatment protocol and the rituximab regimen
• A randomised trial of adequate sample size to distinguish whether the two-dose or four-dose regimen ± cyclophosphamide is effective at achieving an effective B-cell depletion and a favourable clinical response
• Determine an appropriate time to retreat
• Using a standard rituximab regimen would allow for a better comparison between trials
Standardising concomitant therapy
• Classify a change in concomitant immunosuppressant therapy >25% above baseline as partial failure and >50% as complete failure
• Define an increase in the dose of prednisolone >7.5 mg as partial failure and >30 mg as complete failure
Choosing the right disease activity index
• Choosing an index that is validated and is able to capture organ-specific changes: SLE Responder Index and British Isles Lupus Assessment Group, respectively
Defining the endpoints
• Define practically achievable primary endpoints, based on a pilot study and/or taking into account the predicted failure rate for the define cohort, which would detect even small therapeutic benefit
• Define both clinical and nonclinical parameters in the secondary endpoints
• Assess steroid-sparing effect. For example, allow only low-dose prednisolone <10 mg/day and any clinical requirement to increase the dose by >50% as partial failure and >100% as complete failure
Duration of follow-up
• The duration of follow-up should be defined to allow capture of both early and late effects including both safety and efficacy of the therapeutic intervention.
• Defining the adverse events
The reporting of adverse events could be standardised adhering to the OMERACT-recommended guidance [63]

### Patient selection and sample size

From a clinical trial design point of view, there are important differences in the patient cohort, the treatment regimen and the outcome measures used in open studies and real clinical experience when compared with the DBRCTs.

Firstly, the patient cohort in open studies and in clinic experience, at the time of rituximab treatment, had moderate-to-severe disease activity and most had failed conventional immunosuppressants (standard of care). In contrast, patients participating in the two DBRCTs (EXPLORER and LUNAR studies) had active disease, but patients who had failed conventional therapy (cyclophosphamide and calcineurin inhibitors) were excluded. Further, patients with central nervous system manifestations and severe organ-threatening conditions were excluded - situations in which rituximab has demonstrated a favourable record in the open studies [28,45-47]. Capturing the variability in organ-specific outcomes for different interventions tested is important. For example, rituximab may be a better choice than other conventional immunosuppressants when both renal and haematological abnormalities co-exist. A favourable clinical response is more likely in seropositive patients. However, we have previously noted that anti-Sm positivity and/or a low C3 level at the time of treatment is associated with a reduction in the likelihood of sustained benefit from B-cell depletion, and again suggest there is much work to be done to understand lupus disease and factors that may influence the design, population and, ultimately, the outcome of clinical trials [48].

### The therapeutic agent and the regimen

Rituximab has been mainly been used to achieve B-cell depletion in two regimens, either as two doses of 1,000 mg given 2 weeks apart (two-dose regime, commonly used in SLE and RA) or as four doses of 375 mg/m^2 ^(four-dose regime, most common regime used in lymphoma, paediatric autoimmune diseases) given 1 week apart (ocrelizumab in SLE moved on from this to initial doses 2 weeks apart followed by a single infusion every 4 months to achieve and sustain B-cell depletion). Notably, a systematic review of the clinical experience of rituximab for the treatment of refractory SLE suggests that the lymphoma regimen (four doses, 375 mg/m^2^, given 1 week apart) may be more effective in achieving an improvement in disease than the two-dose regimen (two doses given 2 weeks apart) [49]. Based on this review alone, however, it is difficult to draw firm conclusions about the relative efficacy of either regimen. Catapano and colleagues, using both regimens of rituximab for the treatment of refractory SLE, although not in a formal comparative setting, did not detect a significant difference in either the degree of B-cell depletion or clinical outcomes [19]. The two-dose regimen, more convenient for patients requiring just the two hospital infusion visits, is therefore preferred.

Defining standard treatment used in the comparative arm is important, because not doing so would allow generous use of other immunosuppressants - particularly corticosteroids, which are highly effective but associated with unacceptable adverse effects in the long term, not necessarily identified in clinical trials with short-term follow-up.

It would be interesting to take a treatment-to-target approach to achieve an adequate degree of B-cell depletion and clinical response. For example, evidence suggests that the efficacy depends on the extent of B-cell depletion in RA [50]. Several research groups have noted that the degree of B-cell depletion is variable in SLE and that early repopulation is common in patients with a poor response to rituximab [35]. The underlying reasons for the variability in B-cell depletion remain elusive. A polymorphism in Fcγ receptor IIIa has been shown to be important in achieving an adequate degree of B-cell depletion, in favour of the high-affinity genotypes Fcγ receptor IIIa V158F (V, valine; F, phenylalanine) [51]. Treatment-to-target would therefore seem a rational approach to take in an attempt to improve the major clinical response. However, some patients will probably require more frequent doses than others. One approach could be to counterbalance this variation using alternative dose regimes; for example, using two 500 mg doses given 2 weeks apart, as in a recent trial in RA that reported equal efficacy, safety and tolerability between the two regimes using 500 mg or 1 g, provided adequate depletion was achieved [50,52]. Different dosing regimens could potentially have considerable implications: first, patient convenience, with a four-dose regimen requiring more hospital visits; second, a very-low dose regimen has been associated with the development of anti-drug antibodies in SLE while a medium dose (500 mg rituximab ×2) has been shown to be adequate in a number of patients with RA [50]; and, finally, cost-effectiveness of BCDT. In this respect, it has been noted that rituximab might be rapidly consumed in some patients, more frequently in SLE than RA [53]. This consumption would consequently reduce serum rituximab levels and may reduce clinical efficacy.

Taking experience from ocrelizumab therapy in lupus, careful consideration is also necessary when designing studies to test the safety and efficacy of B-cell-targeted approaches, including depletion in patients with active disease also taking mycophenolate. A combination of ocrelizumab and recently commenced mycophenolate does not appear to result in a meaningful additive response and results in an increased risk of infection adverse events (whether the combined impact on the B-cell population of anti-CD20 and mycophenolate was a contributory factor is not understood), whereas this was not the case when used in combination with the EUROLUPUS cyclophosphamide followed by azathioprine regimen.

Defining the standard of care in the placebo arm is important to allow detection of the efficacy for the intervention tested. For example, in the placebo arm a patient with disease activity requiring >7.5 mg prednisolone being classed as a failure will allow detecting the steroid-sparing effect of the intervention, a major advantage in the long term. The question has been raised as to whether to use rituximab in combination with cyclophosphamide, azathioprine or mycophenolate, but there are some conflicting data [19,54]. The definitive answer is therefore awaited.

Another conundrum not yet fully resolved is whether there really is added benefit in using repeated rituximab infusions on a regular basis (that is, maintenance therapy) or whether it is preferable to repeat B-cell depletion only when the patients relapse. A concern about repeated infusion is the potential occurrence of hypogammaglobulinaemia. Information from studies in patients with RA (J Edwards, personal communication) suggests that many patients begin to drop their IgG levels after annual rituximab infusions, particularly in patients with low baseline IgG levels [55]. Comparative data for SLE patients are awaited.

### Clinical evidence for rituximab use - early disease or chronic refractory disease?

Limited evidence from two studies is worth considering. Firstly, as discussed, when used early in conventional immunosuppressive naïve disease, rituximab seems to be effective and has a steroid-sparing effect [24]. Further, Pepper and colleagues have prospectively analysed the response to rituximab for biopsy-proven lupus nephritis, where a total of 14/18 (78%) patients achieved a complete or partial remission with a sustained response in 12/18 at 1 year (67%), with two patients having a relapse with an increase in proteinuria. There was a reduction in prednisolone usage from a mean of 10 mg to 5 mg at 2 years, six patients stopped, six patients managed to reduce the dose and the remaining were maintained on the same dose. Five patients required a temporary increase for extra-renal manifestations [56].

### Defining the outcome measures and clinical response

Clinical outcome measures are to be defined based on evidence, taking into account the probability of detecting change given the expected natural progress of the organ-specific disease manifestations in an appropriate time-frame (potentially in contrast to the artificial time points used in clinical trials). In parallel, it is important to include the biomarkers that predict disease activity and outcomes in SLE. For example, there are a few validated outcome measures that predict end-stage renal disease; it has been shown that doubling of serum creatinine [57,58] and persistently elevated serum creatinine at 48 weeks [58] is predictive of end-stage renal disease. Another routinely available biomarker in clinical practice is urinary protein and an improvement in proteinuria at 1 year [59] and a decrease in serum creatinine or proteinuria at 6 months [60], whilst it may also be reasonably expected that renal response may continue to improve beyond the first year of treatment and may be relevant to consider when identifying the maximal treatment difference for a clinical trial. However, there is limited evidence of reliable predictors of long-term outcome for nonrenal SLE. For reasons discussed earlier, steroid-sparing effect is an important factor when deciding the immunosuppressant of choice [56].

### What disease assessment index to use?

Disease activity indices have been developed with a view to assess either disease activity or damage. The proposed SLE Responder Index, although used in the belimumab studies [61,62], has never been validated or shown to be reliable or sensitive to change or appropriate for wide use when evaluating efficacy with other investigational agents. The key problem with global score indices is that they do not capture partial improvement and/or deterioration.

The definitions of treatment failure and flare remain variable between studies, which limit direct comparison of efficacy of different therapeutic agents. To facilitate a better comparison between studies, therefore, it is important to standardise the definition of a flare and treatment failure.

### Adequate follow-up period to detect significant change in the disease activity and disease damage

Allowing an adequate follow-up period to detect clinically meaningful effects is very important. For example, haematological abnormalities such as anaemia and autoimmune thrombocytopaenia and skin changes such as vasculitic rash improve rapidly; in contrast, response in nephritis may take much longer to detect. Other important factors such as the effects of long-term accruement of organ damage and drug-related adverse effects could only be detected after many years.

### Defining the adverse effects

Adverse events recorded in the clinical trials in SLE have not been adequately standardised to allow comparison between trials. In chronic disease such as SLE where a number of treatments have proved to have modest efficacy, adverse effects associated with treatment have a significant influence on the choice of treatment. As discussed, achieving primary and secondary endpoints of efficacy at the expense of unacceptable adverse events has proven unfruitful in the case of the anti-CD20 (ocrelizumab) in RA [39] whilst the BELONG lupus nephritis trial was stopped early due to an imbalance of infectious adverse events. This finding does raise the question of whether the screening and monitoring criteria can be applied more stringently for the detection of risk or actual opportunistic infections prior to inclusion in the study, particularly when recruiting patients residing in areas endemic for opportunistic infections as mycobacteria or hepatitis. Also, another important question remaining unanswered is whether the adverse effects of biological agents are influenced by other identifiable factors such as disease history and treatment as well as a patient's immunology or indeed ethnicity. A robust definition of categories of adverse events therefore needs to be tested in clinical trials to understand and compare the safety of interventions in clinical trials. For example, is mycophenolate safe to use following rituximab induction therapy? Does the dose of mycophenolate need to be modified to a low-dose regime or should an alternative less potent immunosuppressant such as azathioprine be used? Further, the dose of drug may be better adjusted based on patient characteristics; for example, a dose defined by the weight of the patient rather than a predefined dose (that is, 2 to 3 g). This factor is especially important when considering the use of mycophenolate in patients with low body mass index; for these patients, even 2 g may be a relatively high dose, especially when used in the maintenance regime following rituximab induction therapy. The recording of adverse events in clinical trials and open studies could be standardised adhering to rheumatology-specific criteria such as the OMERACT [63].

## Key messages

• B-cell depletion with rituximab continues to be used in clinical practice for the treatment of refractory SLE, on the basis of a considerable number of publications describing the safety and efficacy data from small open studies and clinical experience whilst noting that it has not been approved by health authorities for the treatment of lupus.

• Contributing features that may have led to the failure of DBRCTs with anti-CD20-mediated B-cell depletion or at least identifying any true treatment effect size probably include concomitant use of high-dose steroids, stringent and nonorgan-specific clinical response criteria, too short a follow-up, and, from a statistical perspective, the sample size. However, the trials confirm the safety of repeated treatment with rituximab.

• A better response to rituximab detected in patients of African-American and Hispanic ancestry highlights the importance of preplanned subgroup analysis and the need to better understand the potential disease drivers of a treatment effect when compared with a standard-of-care regimen in a trial setting.

• The significant biological effects seen with rituximab need to be monitored to assess clinical benefit and risk in the long term.

• Future clinical trial design in SLE and lupus nephritis may be guided by the key working groups of experts, including the European League Against Rheumatism task force, in order to achieve standardisation and to continually apply lessons from both clinical and trial experience.

## Abbreviations

BAFF: B-cell activating factor belonging to the TNF family; BCDT: B-call depletion therapy; BILAG: British Isles Lupus Assessment Group; DBRCT: double-blind: randomised: placebo-controlled trial; dsDNA: double-stranded DNA; mAb: monoclonal antibody; RA: rheumatoid arthritis; SLE: systemic lupus erythematosus.

## Competing interests

DJ has received research grants from Roche/Genentech and Viforpharma, and consulting fees from BIOGEN, Boehringer, GSK, Roche/Genentech and UCB. DC is an employee of MedImmune Ltd and a former employee of Roche Products Ltd. DI has consulted for Roche, asking that his fee is donated to a local research charity. VR declares that he has no competing interests.

## Declarations

This article has been published as part of *Arthritis Research & Therapy *Volume 15 Supplement 1, 2013: B cells in autoimmune diseases: Part 2. The supplement was proposed by the journal and content was developed in consultation with the Editors-in-Chief. Articles have been independently prepared by the authors and have undergone the journal's standard peer review process. Publication of the supplement was supported by Medimmune.
